# Impact of COVID-19 social distancing on medical research from the perspective of postgraduate students: a cross-sectional online survey

**DOI:** 10.7717/peerj.13384

**Published:** 2022-05-12

**Authors:** Chen Dong, Zhou Yu, Wei Liu, Yu Zhang, Zhe Zhang, Lei Zhang, Zhiwei Cui, Xiao Fan, Yuhan Zhu, Han Peng, Botao Gao, Xianjie Ma

**Affiliations:** 1 Department of Plastic Surgery, Xijing Hospital, Fourth Military Medical University, Xi’an, Shaanxi, China; 2 Department of Urology, Xijing Hospital, Fourth Military Medical University, Xi’an, Shaanxi, China

**Keywords:** COVID-19, Social distancing, Medical research, Postgraduate students, Online survey, Cross-sectional study

## Abstract

**Objective:**

To investigate the impact of COVID-19 social distancing on medical research from the perspective of postgraduate students.

**Methods:**

A cross-sectional study using an online survey was conducted from October 31 to November 1, 2021. A questionnaire was used to assess the impact of COVID-19 social distancing on medical research among postgraduate students. The questionnaire included basic information, medical research information, and information about social distancing measures. Participants also completed the self-made Research Work Affected Scale of Postgraduates (RWAS-P; qualitative evaluation: very mildly 0–10; mildly 11–20; moderately 21–30; severely 31–40; very severely 41–50). Logistic regression was used to identify factors related to the impact of COVID-19 social distancing.

**Results:**

A total of 468 participants were analyzed; 95.2% of the participants adhered to social distancing measures. The median total RWAS-P score was 22. The median RWAS-P scores for earlier research data, current research projects, future research plans, paper publication, and graduation schedule were 2, 6, 6, 6, and 4, respectively (score range 0–10). The higher grade of students, experimental research, and existence of inappetence or sleeplessness were related to negative attitude towards COVID-19 social distancing (odd ratio = 6.35, 9.80, 2.31, 2.15, 1.95, respectively).

**Conclusions:**

Participants reported that social distancing had a moderate overall impact on their medical research. Social distancing had the greatest impact on current research projects, future research plans, and paper publications among postgraduate students. Higher grade level, experimental research type, inappetence, and sleeplessness were related to the impact of social distancing on their medical research.

## Introduction

As of November 30, 2021, the ongoing coronavirus (COVID-19) pandemic caused over 260 million infections and approximately 5.2 million deaths worldwide (WHO, 2021) There is an urgent need to stop the spread of COVID-19 to save lives. Social distancing is a non-pharmaceutical measure that reduces physical contact between infectious sources and susceptible people during a disease outbreak (Abdullahi et al., 2020). Faced by the threats posed by COVID-19, most countries rapidly implemented social distancing policies to reduce the transmission (Eubank et al., 2020). Research has confirmed that social distancing successfully reduces the transmission, severity, and number of deaths associated with COVID-19 (Matrajt & Leung, 2020). Regardless, it has been proven that social distancing negatively affects an individual, both physically and psychologically (D’Acquisto & Hamilton, 2020; Rodríguez-Fernández et al., 2021).

In October 2021, a new wave of the COVID-19 outbreak originated in western China and spread to more than twenty provincial-level regions  (CGTN, 2021). To cut off the COVID-19 transmission routes, social distancing measures in Xi’an, Shaanxi Province, China, among other cities, were strengthened.

Medical researchers must cooperate with each other, communicate face-to-face and take part in seminars or workshops regularly. However, adherence to social distancing rules meant limiting the number of researchers in laboratories and suspending or conducting scholarly activities online. As for clinical study, the running of the trials during a pandemic is also affected, for patient’s hesitancy or inability to continue investigative treatments due to self-isolation/quarantine or limited access to public places (AlNaamani, AlSinani & Barkun, 2020). The attitudes towards COVID-19 social distancing affecting medical research among postgraduate students are not yet well understood, and related survey may provide a scientific reference for developing public health policies that balance pandemic prevention and medical research work. Thus, this study’s researchers conducted an online survey to investigate the impact of COVID-19 social distancing on medical research from the perspective of these front-line researchers.

## Materials & Methods

### Study design

This was a cross-sectional online survey among the medical postgraduate student population of the Fourth Military Medical University in Xi’an, Shaanxi Province, China. Data were collected from October 31, 2021 to November 1, 2021 through an online anonymous questionnaire. The online survey platform https://www.wjx.cn/ was used to publish the questionnaire and to generate the uniform resource locator (URL) link for responses; the survey and its response mechanism were easily distributed through WeChat. Respondents visited the URL on their mobile phones to respond to the questionnaire. Invitations to participate in an anonymous survey through WeChat were sent by URL link to 700 students. Of them, 502 (71.7%) responded. Respondent inclusion criteria were individuals who (1) were postgraduate students undertaking medical research work in a medical college, (2) at least 18 years old, (3) able to read and complete the self-administered questionnaire independently, and (4) voluntary participants of the survey. Individuals who could not understand the questionnaire completely were excluded.

### Participants

Due to the functionality of the online survey platform https://www.wjx.cn/, no personal identifiers of the respondents (including names or photographs) were obtained. All participants were over the age of 18. Thus, the survey was anonymous and did not present any potential harm to participants’ physical and mental health. All participants voluntarily participated in this study. Prepared e-survey forms were sent to students *via* a link created for the purpose. In order to fully inform the participants and make the participants have a preliminary understanding of the questionnaire contents, we set a preview mode. The response time was automatically recorded until the participants agreed to participate in the survey and/or end the preview. The respondent students could only proceed to participate in the online survey when they agreed and gave their consent after reading the aim of study and clicking ‘yes’ to the informed consent statement in the first part of the questionnaire.

### Sample size

The initial minimum participant sample size was calculated using PASS 11 software (https://www.ncss.com/software/pass/; NCSS, LLC. Kaysville, UT, USA) (Tang et al., 2021) and based on a two-sided 95% confidence interval (CI) with a width equal to 0.10 when the sample proportion was 0.50. From these calculations a minimal sample size of 402 participants was estimated. Estimating that 20% of questionnaires would be invalid, the recruitment of a minimum of 483 individuals was planned.

### Measures

A concise and structured questionnaire was designed to investigate the impact of COVID-19 social distancing on medical research from the perspective of postgraduate students. The questionnaire mainly included the following four parts: (1) basic information, (2) medical research information, (3) social distancing measure information, and (4) the self-made scale that assessed if research work was affected (Research Work Affected Scale of Postgraduates, RWAS-P; score range 0–50; qualitative evaluation: very mildly 0–10, mildly 11–20, moderately 21–30, severely 31–40, very severely 41–50). A 5-point Likert rating system was adopted within the first three parts. A visual analogue scale (score range 0–10) was used for part four.

Two rounds of expert consultations were conducted to develop the questionnaire including RWAS-P. The selected experts included medical professors, laboratory technologists, senior postgraduate students, statisticians, and linguists. RWAS-P was used to assess if the medical research work of postgraduate students was affected. The questionnaire was initially pilot tested before the final version of the questionnaire was sent.

### Statistical analysis

Descriptive data were presented as mean ± standard deviation, median (minimum, maximum), or frequencies and proportions. The reliability and validity were used to evaluate the RWAS-P including item 16–20. Reliability of the RWAS-P consisting item 16–20 was measured by internal consistency. Cronbach’s *α* and the Guttman split-half coefficients were calculated to test the internal consistency. The RWAS-P was considered highly reliable when the coefficients were higher than 0.70 (Boparai, Singh & Kathuria, 2018). Construct validity was evaluated by factor analysis. To establish if the collected information was suitable for the factor analysis, Bartlett’s test of sphericity and the Kaiser-Meyer-Olkin (KMO) test were performed. When the KMO test value was higher than 0.70 and the *p* value of Bartlett’s test of sphericity was <0.05, the factor analysis proceeded (Limone et al., 2021). The items in RWAS-P were considered acceptable construct validity when factor loading values were 0.5 or above (Limone et al., 2021). The groups whose medical research was affected by social distancing were categorized based on RWAS-P median score. Subsequently, logistic regression was employed to identify possible factors affecting the research of medical postgraduates. Significance of the univariate model at the level of *P* < 0.10 was considered for entry into a multivariate model. The criterion to remain in the model was *P* < 0.05. For the regression, odds ratio (OR) and the respective 95% CI were estimated. All analyses were performed using SPSS 25.0 (IBM Corporation, New York, NY, USA).

## Results

### Contents of the questionnaire

After screening by experts, 21 items were included in the final questionnaire (Table 1). Six items related to basic information about participants; five items investigated social distancing measures participants undertook; four items inquired about type, progress, and expectations of participants’ medical research; and five items were RWAS-P items for subjective evaluation. One last item for quality control required participants to answer whether they understood the meaning of the above items completely. Responses were required for all questionnaire items; incomplete questionnaires could not be submitted.

**Table 1:** Main contents of the questionnaire^a^.

Items
**Part 1. Basic information**
(1) What’s your gender? [single choice]
A. Male; B. Female
(2) How old are you? [completion, integers needed]
(3) What’s your marital status? [single choice]
A. Unmarried; B. Married; C. Others
(4) What’s your degree intended to apply and student year? [single choice]
A. Master (first year); B. Master (second year); C. Master (third year or more);
D. Doctor (first year); E. Doctor (second year); F. Doctor (third year or more);
(5) Your personal average monthly income last year (including social benefits, living expenses from other people, etc.)? [single choice]
A. <2000 RMB; B. 2000–5000 RMB; C. 5000–10000 RMB; D. ≥10000 RMB
(6) Do you have a normal job half time? [single choice]
A. Yes; B. No
**Part 2. Medical research related information**
(7) Which of the following statements matches the general type of your medical research? [single choice]
A. Laboratory research; B. Clinical research; D. Others
(8.1) (If answer in item 7 was A, please answer this question) Which of the following statements matches the specific type of your medical research? [single choice]
A. *In vitro*; B. *In vivo*; C. *In vitro* and *in vivo*; D. Others
(8.2) (If answer in item 7 was B, please answer this question) Which of the following statements matches the specific type of your medical research? [single choice]
A. Randomized Controlled Trial; B. Prospective cohort study; C. Respective cohort study;
D. Case-control study; E. Cross-sectional study; F. Others
(9) What is the highest SCI impact factor you expect for your paper of graduate research? [single choice]
A. None or <1; B. 1–3; C. 3–5; D. 5–10; E. ≥10
(10) In your opinion, what is the percentile of your current research progress? [single choice]
A. 0; B. 10%; C; 20%; D; 30%; E. 40%; F. 50%; G. 60%; H. 70%; I. 80%; J. 90%; K. 100%
**Part 3. Social distancing measures related information**
(11) In the most recent week of social distancing, which of the following areas was your main social activity site? [single choice]
A. Home; B. Workplace and living area; C. Community; D. Public place.
(12) In the most recent month of social distancing, how long does the current social distancing measures you take? [single choice]
A. 0–3 d; B. 4–7 d; C. 8–14 d; D. ≥15 d
(13) In period of social distancing, whether you continue your medical research remotely or receive guidance from others remotely? [single choice]
A. Never; B. Rarely; C. Sometimes; D. Often; E. Always
(14) In the most recent week of social distancing, how about your sleep? [single choice]
A. Very poor; B. Poor; C. General; D. Good; E. Very good
(15) In the most recent week of social distancing, how about your appetite? [single choice]
A. Very poor; B. Poor; C. General; D. Good; E. Very good
**Part 4. Research Work Affected Scale of Graduates (RWAS-P; a 0–10 visual analog scale to measure the intensity of effect; 0 = barely, 10 = extremely)**
(16) Social distancing makes your earlier research data be damaged or loosed.
(17) Social distancing makes your current research projects be postponed.
(18) Social distancing will make your future research plans to be adjusted.
(19) Social distancing will make the publication of your research paper to be delayed.
(20) Social distancing will disrupt your original graduation schedule.
**Part 5. Quality control**
(21) Do you totally understand the meanings of all questions above? [single choice]
A. Yes, I do; B. No, I have any confusion about the questionnaire.

**Notes.**

a Translated from Chinese.

### The reliability and validity of the RWAS-P

Cronbach’s *α* and the Guttman split-half coefficients were 0.92 and 0.89, respectively. The Bartlett’s test of sphericity chi-square statistic was 1967.91 (*P* < 0.001), and the KMO value was 0.85. Only one component was extracted with 76.43% of the total variance, which is consistent with the single-dimensional design for the RWAP-S. Factor loadings of item 16–20 in the RWAP-S were 0.926, 0.917, 0.894, 0.853, and 0.772, respectively. The above results demonstrated the well-performed reliability and construct validity of the RWAS-P.

### Participant characteristics

**Figure 1:**
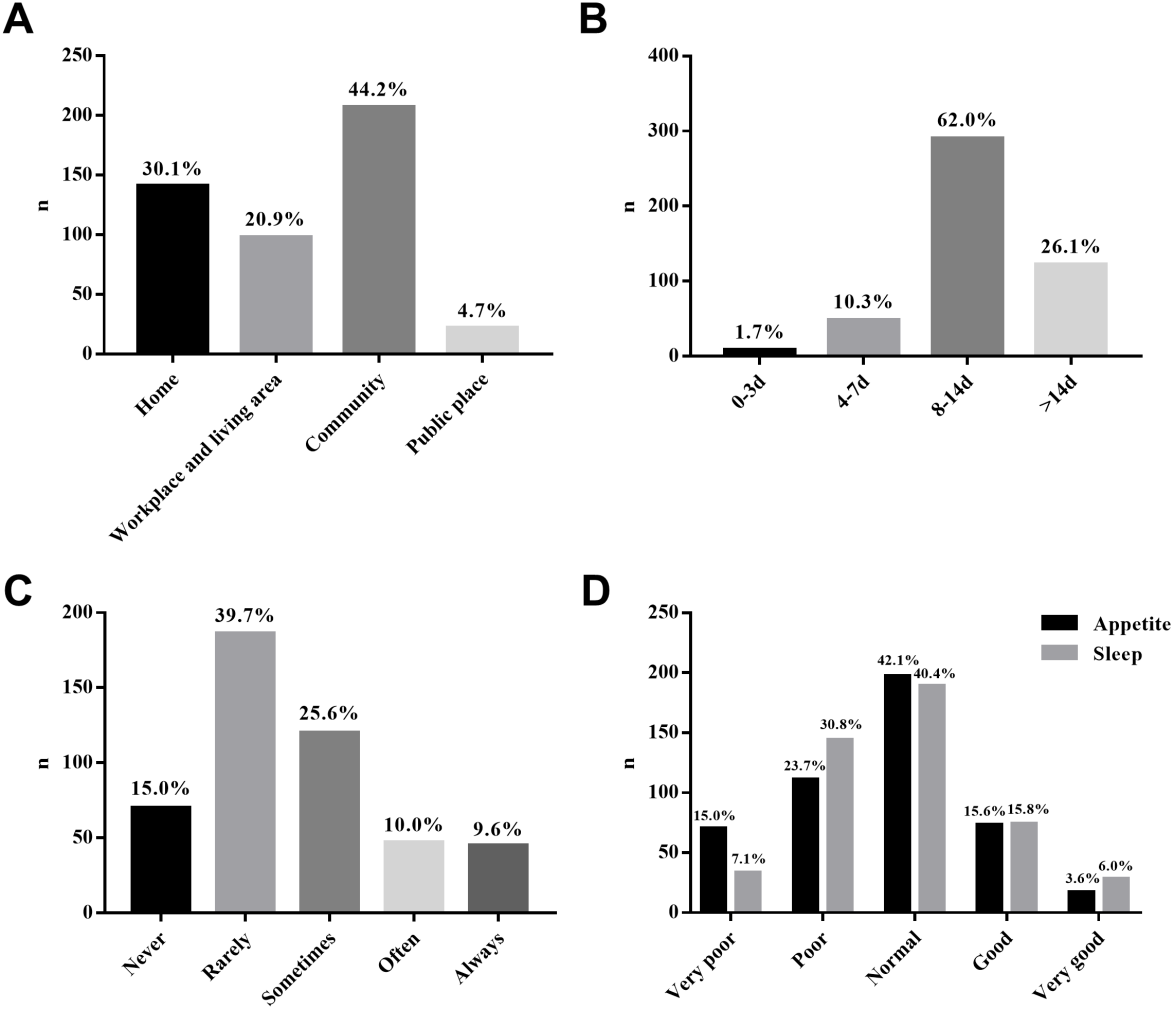
Descriptive results related to social distancing measures. SA, social activities. (A) Social activity areas; (B) social distancing duration; (C) remote assistance; (D) conditions of sleep and appetite.

A total of 502 respondents were recruited for this survey; 468 questionnaires were considered valid (effective rate = 93.2%). The median age of participants was 26 (20, 40) years. The proportion of male and female participants was 54.1:45.9. A total of 150 (32.1%) participants were married. The ratio of participants applying master’s degree and doctoral degree in the survey was 80.6:19.4. Postgraduate student participants in their first, second, and third years or beyond were 170 (36.3%), 126 (26.9%), and 172 (36.8%), respectively. Laboratory researchers represented 56.0% (262/468) and clinical researchers represented 35.5% (116/468) of the sample. Results related to social distancing measures are shown in Fig. 1.

### Factors associated with effects on postgraduate medical research

The median total RWAS-P score was 22 (score range 0–50; qualitative evaluation: very mildly 0–10; mildly 11–20; moderately 21–30; severely 31–40; very severely 41–50). Using 22 as the cutoff value for participants in the group (*n* = 258) with medical research less affected by the social distancing, other participants were assigned to the group (*n* = 210) with medical research more affected by the social distancing. The details of these two groups and the results of univariate logistic regression are presented in Table 2. The results of multivariate logistic regression were showed in Table 3.

**Table 2:** Differences between the less and the more affected groups.

Factors	Affected group^a^, *n* (%)		*p* value
	Less (*n* = 258)	More (*n* = 210)	
Gender			
Male	129 (50.0)	124 (59.0)	1 (reference)
Female	129 (50.0)	86 (41.0)	0.051
Age, years			
<24	60 (23.3)	16 (7.6)	1 (reference)
24–27	85 (32.9)	75 (35.7)	<0.001
27–30	57 (22.1)	51 (24.3)	<0.001
30–33	35 (13.6)	41 (19.5)	<0.001
≥33	21 (8.1)	27 (12.9)	<0.001
Married status			
Unmarried	187 (72.5)	131 (62.4)	1 (reference)
Married and others	71 (27.5)	79 (37.6)	0.020
Year of students			
First-year	148 (57.4)	22 (10.5)	1 (reference)
Second-year	51 (19.8)	75 (35.7)	<0.001
Third-year and above	59 (22.9)	113 (53.8)	<0.001
Degree intended to apply			
Master	211 (81.8)	166 (79.0)	1 (reference)
Doctor	47 (18.2)	44 (21.0)	0.457
Monthly income, RMB			
<5,000	164 (63.6)	89 (42.4)	1 (reference)
5,000–10,000	62 (24)	84 (40.0)	<0.001
≥10,000	32 (12.4)	37 (17.6)	0.006
Having normal job half time			
Yes	82 (31.8)	86 (41.0)	1 (reference)
No	176 (68.2)	124 (59.0)	0.040
Medical research type			
Laboratory research	130 (50.4)	132 (62.9)	1 (reference)
Clinical research	107 (41.5)	59 (28.1)	0.003
Others	21 (8.1)	19 (9.0)	0.734
Expected SCI impact factor			
None or <1	59 (22.9)	56 (26.7)	1 (reference)
1–3	60 (23.3)	44 (21.0)	0.344
3–5	72 (27.9)	54 (25.7)	0.364
≥10	67 (26)	56 (26.7)	0.625
Current research progress			
<20%	127 (49.2)	29 (13.8)	1 (reference)
20–50%	64 (24.8)	69 (32.9)	<0.001
50–80%	42 (16.3)	84 (40.0)	<0.001
≥80%	25 (9.7)	28 (13.3)	<0.001
Social distancing intensity			
Mild (Public place)	16 (6.2)	6 (2.9)	1 (reference)
Moderate (Community)	128 (49.6)	79 (37.6)	0.319
Severe (Workplace and living area)	44 (17.1)	54 (25.7)	0.023
Very severe (Home)	70 (27.1)	71 (33.8)	0.050
Social distancing duration			
<7d	31 (12.0)	25 (11.9)	1 (reference)
7–14 d	161 (62.4)	129 (61.4)	0.982
≥14	66 (25.6)	56 (26.7)	0.876
Remote assistance			
Less	141 (54.7)	115 (54.8)	1 (reference)
Moderate	65 (25.2)	55 (26.2)	0.869
More	52 (20.2)	40 (19.0)	0.811
Inappetence			
No	187 (72.5)	100 (47.6)	1 (reference)
Yes^b^	71 (27.5)	110 (52.4)	<0.001
Sleeplessness			
No	184 (71.3)	107 (51.0)	1 (reference)
Yes^b^	74 (28.7)	103 (49.0)	<0.001

**Notes.**

a The median of 22 is set to a cutoff value.

b Answers were *poor* or *very poor* in item 14 or 15 of the questionnaire.

**Table 3:** Multivariate logistic regression analysis of factors associated with the social distancing impacts on medical research of postgraduates^a^.

Factors^*^	*β*	OR (95% CI)	*p* value
Second-year students vs. first-year students	1.85	6.35 (3.19–12.64)	<0.001
Third-year and above students vs. first-year students	2.28	9.80 (4.03–23.83)	<0.001
Medical research type, laboratory vs. clinical research	0.84	2.31 (1.41–3.78)	0.001
Inappetence, yes vs. no	0.76	2.15 (1.33–3.47)	0.002
Sleeplessness, yes vs. no	0.67	1.95 (1.20–3.16)	0.007

**Notes.**

a The median of 22 is set to a cutoff value.

* Only factors with statistical significance were listed in the table.

Abbreviations OR odds ratio CI confidence interval

### Sub-analysis of each RWAS-P item

The median single scores of earlier research data, current research projects, future research plans, paper publication, and graduation schedule from the RWAS-G were 2, 6, 6, 6, and 4 respectively (score range 0–10). These cutoff values were used to divide participants into two groups: a less affected group and a more affected group. The primary sub-analysis results for each item in the RWAS-P are shown in Table 4.

**Table 4:** Sub-analysis for each RWAS-P item^*^.

Factors	Evaluation purpose of items, OR (95% CI), *p* value				
	Earlier research data	Current research projects	Future research plans	Paper publication	Graduation schedule
Gender, male vs. female	0.56 (0.35–0.89), 0.015	–	–	–	–
Second-year students vs. first-year students	5.03 (2.54–9.96), <0.001	6.86 (3.49–13.50), <0.001	6.72 (3.41–13.27), <0.001	5.23 (2.69–10.16), <0.001	5.47 (2.64–11.33), <0.001
Third-year and above students vs. first-year students	6.94 (3.65–13.20), <0.001	6.64(2.75–15.99), <0.001	9.07 (3.67–22.39), <0.001	9.62 (3.98–23.24), <0.001	8.70 (3.57–21.22), <0.001
Degree intended to apply, master vs. doctor	–	–	–	–	2.68 (1.46–4.90), 0.001
Medical research type, experimental vs. clinical research	–	2.99 (1.80–4.98), <0.001	2.89 (1.72–4.85), <0.001	1.93 (1.19–3.15), 0.008	–
Current research progress, 50–80% vs. <20%	–	2.61 (1.04–6.53), 0.040	–	–	–
Inappetence, yes vs. no	1.74 (1.08–2.81), 0.024	–	1.88 (1.13–3.13), 0.015	–	1.87 (1.16–3.00), 0.031
Sleeplessness, yes vs. no	2.25 (1.39–3.64), 0.001	1.83 (1.14–2.93), 0.012	1.97 (1.19–3.28), 0.009	1.97 (1.25–3.10), 0.003	2.68 (1.46–2.72), 0.001

**Notes.**

* Only factors with statistical significance were listed in the table.

Abbreviations OR odds ratio CI confidence interval

## Discussion

Postgraduate students participating in this study reported that social distancing had moderate overall impact on their medical research. Current research projects, future research plans, and paper publication were affected the most by social distancing from the perspective of postgraduate students. Higher grade level, laboratory research type, inappetence, and sleeplessness were shown to be related to the negative effects of social distancing.

The COVID-19 global pandemic has significantly impacted many aspects of life (Hemmati & Rastgoftar, 2021). Strong social distancing measures curtailed the rapid rise in COVID-19 cases, deaths, and hospitalizations (Courtemanche et al., 2020; Lyu & Wehby, 2020). However, social distancing itself has had various negative effects on economic activity, daily life, and individual health (Lytras & Tsiodras, 2021). Medical research has also been disturbed by social distancing measures in varying degrees. Despite this, only a few studies have briefly mentioned the adverse effects of social distancing related to COVID-19 on medical research, including postponed essential research and suspended scientific conferences (Wang & Dai, 2020).

To investigate the impact of social distancing on medical research, this study selected postgraduate students as a representative population, because they are active researchers and undertake a significant amount of routine research work. As the amount of medical research affected by social distancing is difficult to quantify with objective indicators, a multidimensional self-rating scale, the RWAS-P, was designed and used to measure these effects. The RWAS-P was found to have sufficient reliability and construct validity, providing a foundation for follow-up analysis.

At the student participants’ affiliated university, most postgraduate students adhered strictly to social distancing rules and policies. These medical postgraduate students consciously abided by the social distancing measures, including physical distancing, lab or workplace closure, and avoidance of mass gatherings. Through mid-November 2021, no medical postgraduate students enrolled in the study’s survey contracted COVID-19. The effectiveness of social distancing was confirmed by the demonstrated suppression of COVID-19 transmission. More than 80% of the participants reported practicing social distancing for more than seven days a month during the pandemic. Any possible close contact with COVID-19 patients or asymptomatic carriers requires at least a 7-day observation period (Tu et al., 2021). Nearly half of the participants required remote means to continue work or receive guidance from professors/supervisors. A previous survey found that medical postgraduate students benefited from stay-at-home measures as they spent more time on learning or manuscript-writing (Varadarajan, Brown & Chalkley, 2021). However, interaction with peers and in-person discussions were not often possible during the remote learning period. A survey also found that postgraduate students were more likely than undergraduates to experience obstacles when transitioning to remote learning. These obstacles included serving as the caretaker of one or more family members, difficulty concentrating, and increased anxiety and fatigue  (Soria, Chirikov & Jones-White, 2020). These disadvantages also explain why working remotely and receiving guidance did not reduce the negative effects of social distancing measures in the latter analysis.

Moreover, approximately 40% of the participants complained of varying degrees of inappetence and sleeplessness (answering RWAS-P item 14 or 15 with very poor or poor). This demonstrated that social distancing was related to the negative overall psychological condition of some medical postgraduate students. Previous research also found that under harsh COVID-19 social distancing measures, the prevalence of anxiety or depression was approximately 20% among medical students in China (Xiao et al., 2020).

Medical postgraduate students noted that social distancing had a moderate overall effect on their research; the total qualitative evaluation from the RWAS-G was over the intermediate range. Higher grade/level of study, laboratory research, and instances of inappetence or sleeplessness were related to the negative assessment of the impact of COVID-19 social distancing.

Second year students and third year students were 6.35 and 9.80 times more likely to be affected by social distancing than first year, respectively. Compared to first-year postgraduate students, students in their second year and beyond believed that the pandemic was more disruptive (Rana et al., 2020). This difference may attribute to the less pressure to find a job or to complete the thesis required for graduation for first-year postgraduate students (Xiao et al., 2020). Medical postgraduate students who were laboratory researchers were 2.31 times more likely to be affected by social distancing than clinical researchers. With minimal preparation for unplanned lab closures, cell lines in proliferation had to be frozen, mouse colonies in reproduction had to be culled, and ongoing experiments had to be terminated abruptly (Freedman et al., 2020). Although clinical researchers also faced challenges, remote measures, such as telephone and video visits, internet monitoring, and electronic capture of signatures and data, largely offset the negative effects of social distancing (Marcum et al., 2020; Tavazzi, 2021; Valmorri et al., 2021).

The participants with sleeplessness and inappetence were 2.15 and 1.95 times more likely to be affected by social distancing than others, respectively. Disturbances of sleep and appetite were common signs of psychological discomfort during the COVID-19 pandemic (Birmingham et al., 2021; Verma, 2020). Participants experiencing sleeplessness and inappetence were inclined to make more negative evaluations of the effects of social distancing. These participants may have faced increased anxiety, feelings of loneliness, or depression (Lee et al., 2021; Medeiros et al., 2020).

According to the RWAS-P scores, current research projects, future research plans, and paper publication were most affected by social distancing from the perspective of postgraduate students. Sub-analysis revealed that the female medical postgraduate population experienced less damage or loss of earlier research data than their male counterparts. Additionally, researchers concluded that doctoral degree applicants were more concerned with delayed graduation, as this would affect their eligibility.

The study results lead to the following recommendations: (1) When there is no pandemic, medical postgraduate students should reserve time in their schedules for unexpected interruptions and prepare for executable alternatives to research. (2) In terms of the research that must be maintained during the pandemic, school or laboratory managers should try their best to guarantee researchers to conduct study in closed and secure environments. The closed-loop management can protect researchers and at the meantime ensure the continuity of research even if a COVID-19 outbreak occurs. If safety cannot be guaranteed, it should be suspended in time. (3) When the pandemic circumstances abate, laboratory managers should prioritize resuming the research work of postgraduate students close to graduation—senior postgraduate students and doctoral degree applicants. (4) Data copying and collation of research work should be kept up to date to prevent any loss of experimental data in the case of sudden laboratory/workplace closures. (5) Some medical postgraduate students require psychological help to mitigate the negative mental health effects of the social distancing measures. Appropriate action plans are needed.

COVID-19 social distancing has had a moderate effect on the medical research of postgraduate students. However, adherence to public health measures to end the pandemic as quickly as possible is necessary for medical research in the long run.

Regarding the research methods, some limitations must be acknowledged. First, the study was conducted with participants in China, thus it may not be generalizable to other countries. Second, because the study was cross-sectional, causality cannot be determined. Third, the study was conducted in one medical school, and the basic situations of students in this school may differ from other ones. However, social distancing measures in China are similar nationally, and the graduate students enrolled in this research institute range from grade one to three years as well as from master’s degree students to doctoral degree students, and these students come from different places in China. Thus, the study findings are more extensive representative of medical postgraduate students in China to some extent. For areas with similar social distancing measures, the results of the study can be also used as a reference for public health policy development.

## Conclusions

The impact of COVID-19 social distancing on medical research from the perspective of postgraduate students have rarely been reported. Postgraduate students participating in this study reported that social distancing had a moderate overall effect on their medical research. Current research projects, future research plans, and paper publication were affected the most by social distancing from their perspective. Higher grade level, laboratory research type, inappetence, and sleeplessness were shown to be related to the negative effects of social distancing. However, adherence to public health measures to end the pandemic as quickly as possible is necessary for medical research in the long run. Moreover, this article fills a known literature gap related to COVID-19 transmission-reduction efforts and the resulting impact on postgraduate students and their research projects. The RWAS-P tool is a novel contribution for quantitative evaluation of impact on postgraduate students’ research work. As the COVID-19 situation continues, it is necessary to investigate the effects of social distancing on medical research.

##  Supplemental Information

10.7717/peerj.13384/supp-1 Table S1 Raw data All results of the online survey.

## Additional Information and Declarations

### Data Availability

The following information was supplied regarding data availability: The raw measurements are available in the Supplementary File.
